# The reliability and validity of the Norwegian version of the Victorian Institute of Sports Assessment for gluteal tendinopathy questionnaire (VISA-G-Norwegian) for patients with greater trochanteric pain syndrome

**DOI:** 10.1186/s12891-023-06901-0

**Published:** 2023-09-29

**Authors:** Håkon Sveinall, Per Kristian Wenstad, Angela M. Fearon, Gjermund Skyttemyr, Elisabeth Thornes, Heléne Engberg Skaara, Niels Gunnar Juel, Jens Ivar Brox, Cecilie Roe, Marianne Bakke Johnsen

**Affiliations:** 1 Department of Physical Medicine and rehabilitation, Oslo University Hospital, Oslo, Norway; 2 Department of Rehabilitation Science and Health Technology, Faculty of Health Sciences, Oslo Metropolitan University, Pilestredet 44, Oslo, 0167 Norway; 3 University of Canberra Research Institute Sport and Exercise, Faculty of Health, University of Canberra, Canberra, Australian Capital Territory Australia; 4 Trauma and Orthopedic Research Unit, Canberra Hospital, Canberra, Australian Capital Territory Australia; 5 Martina Hansens Hospital, Sandvika, Norway; 6 Department of General Practice, University of Oslo, Oslo, Norway; 7 Faculty of Medicine, Institute for Clinical Medicine, University of Oslo, Oslo, Norway

**Keywords:** Greater trochanteric pain syndrome, Hip pain, Lateral hip pain, Bursitis, Tendinopathy, PROM, Reliability, Validity

## Abstract

**Background:**

Greater Trochanteric Pain Syndrome (GTPS) is a common chronic musculoskeletal condition that may affect physical function, quality of life and sleep. The Victorian Institute of Sport Assessment-Gluteal questionnaire (VISA-G) has been developed as a Patient-Reported Outcome Measurement (PROM) to address pain, everyday activities, physical activities, and difficulty with weight bearing activities. The aim of the study was to test the reliability, validity and floor and ceiling effects of the Norwegian version of the VISA-G (VISA-G-Norwegian) in a population with GTPS in a specialist health care setting.

**Methods:**

This psychometric evaluation of the VISA-G-Norwegian questionnaire were conducted with a prospective observational design. The VISA-G was translated into Norwegian following recommended guidelines. A subgroup repeated the VISA-G-Norwegian a week after the initial submission. For the reliability, the Intraclass Correlation Coefficient (ICC_2.1_), Standard Error of the Measurement (SEM) and the Smallest Detectable Change (SDC_95%_) were calculated. Internal consistency was measured using a Cronbach´s alpha. Floor and ceiling effects were evaluated, and construct validity was assessed with three a priori hypotheses.

**Results:**

78 participants were included in the study of which 47 stable participants undertook the test-retest reliability arm of the study. The ICC_2.1_ for the total score was 0.85 (95% CI 0.68, 0.92), SEM was 6.6 points and SDC_95%_ 18.4 points. Cronbach`s alpha was 0.77 (95% CI 0.69, 0.84). No floor or ceiling effects were found in the total score, but ceiling effect was found in three of the eight items. For construct validity, one of the three hypotheses were confirmed. VISA-G-Norwegian correlated to the modified Harris Hip Score (mHHS), Oswestry Disability Questionnaire (ODI) and Numeric Pain Rating Scale (NPRS), 0.64, -0.75 and − 0.63 respectively.

**Conclusion:**

The VISA-G-Norwegian has acceptable reliability and validity, despite ceiling effect of individual items. The large SDC_95%_ should be considered when measuring change in similar cohorts with GTPS. For a potential future version, it would be recommended to consider response options for questions with ceiling effect and the comprehensibility of question eight.

**Trial registration:**

Registered at ClinicalTrials.gov the 28/02/2020 (NCT04289922).

## Introduction

Greater Trochanteric Pain Syndrome (GTPS) is a common chronic, painful, and disabling musculoskeletal condition known to affect physical function, quality of life and sleep [1–5]. It is considered an unspecific condition, and patients may have a variety of physical and psychological symptoms that influence participation in social activities. Different diagnostic labels have been used to describe the condition, for example lateral hip pain. The condition primarily affects middle-aged women (45–63 years old), at a ratio females:males of 4:1 [3, 6]. The prevalence and incidence are reported from 4.2 to 3.3 per 1000 person-year, respectively, in primary care [7, 8]. Amongst women and men with knee pain, the prevalence is reported up to 23.5% and 9.5%, respectively [9], and 20–35% in those with low back pain [10, 11]. Other risk factors for developing GTPS are obesity and lower femoral neck angle [3, 6].

A Patient-Reported Outcome Measure (PROM) evaluates a patient’s health status. Questionnaires are commonly applied to acquire PROM data [12]. The Victorian Institute of Sport Assessment - Gluteal questionnaire (VISA-G) has been developed as a condition specific PROM in accordance with the COnsensus-based Standards for the selection of health Measurement INstruments (COSMIN) recommendations, to evaluate the degree of severity of disability associated with GTPS [1, 12]. A recent systematic review found that there exists moderate quality evidence of sufficient construct validity, and low quality evidence of sufficient reliability and measurement error for the VISA-G [13]. Still, they concluded that The VISA-G is the preferred available option to capture the disability associated with gluteal tendinopathy [13]. Lately, the VISA-G has been adapted into different languages with even higher psychometric characteristics compared with the original version [14–18]. However, several of the previous studies have included below the recommended sample size of fifty to analyze the reliability and validity by the COSMIN checklist [19]. Only the Turkish, Brazilian, and French studies have an acceptable sample size with 108, 68 and 52 patients respectively [14, 17, 18].Thus, further testing with an adequate sample size is required. So far, only the Turkish study has investigated the VISA-G questionnaire on patients with GTPS in a specialist health care setting [18]. Collecting psychometric properties of questionnaires in different populations is necessary as the reliability and validity may vary from one population to another. Finally, no previous study has investigated the floor and ceiling effect on individual items. Thus, the aim of the study was to test the reliability, construct validity, and floor and ceiling effects of the Norwegian version of the VISA-G (VISA-G-Norwegian) with an adequate sample size of more than 50 in a population with GTPS in a specialist health care setting.

## Methods

This cross-cultural adaption and psychometric evaluation of the VISA-G-Norwegian questionnaire were conducted with a prospective observational design, and in accordance with the COSMIN checklist [19].

**Translation and cross-cultural adaption**. Permission to translate and culturally adapt the VISA-G questionnaire was obtained from the original developer Dr. Angie Fearon (A.F.) [1]. The original questionnaire was translated into Norwegian following recommended guidelines in August 2018 [20]. Firstly, three translators with Norwegian as their first language translated the original English version into Norwegian. Where one of the translators was naïve to patients with GTPS and the two other translators had expertise in these patients, one medical doctor and one physiotherapist. The two physiotherapists who led the translation (H.S. and M.B.J.) synthesized the three versions into one. Discrepancies between the three versions were compared and the option with most agreement was chosen, if all three had different translations, H.S. and M.B.J. made a final judgement on which version should be retained. Further, one professional translator- and one Norwegian speaking with English as her first language, both naïve to GTPS, translated the synthesized version back into English. At this stage, H.S. and M.B.J. met the original developer (A.F.) which constituted a committee, approving the back translated versions. A pre-final version was then presented to 10 GTPS patients to evaluate comprehensibility and relevance, the patients were asked to report any difficulties responding to the questionnaire, which was discussed in the translation group. A minor amendment in the layout was made from the pre-final to the final version.

**Participants**. Eligible patients with GTPS, referred to the physiotherapy outpatient clinic at The Department of Physical Medicine and Rehabilitation at Oslo University Hospital and The Orthopedic Department at Martina Hansens Hospital, were included as a part of their treatment. The inclusion criteria were ≥ 2/5 positive tests for GTPS and ≥ 18 years old. A physiotherapist clinically assessed patients for inclusion, patients underwent a clinical test battery for GTPS, consisting of pain on palpation of the greater trochanter, single leg stance test, Flexion Abduction External Rotation (FADER), Flexion Adduction External Rotation with Resistance (FADER-R) and Adduction with Resistance (ADD-R) [21]. The exclusion criteria were not being able to read or write Norwegian.

This study aimed to include 100 participants, as indicated as “very good” to perform analysis for reliability, internal consistency and construct validity by The COSMIN study design checklist [19].

**Patient reported-outcome measures**. At baseline, the participants answered a set of sociodemographic variables such as age, sex, educational level, work status, duration of pain and level of physical activity, modified Harris Hip Score (mHHS), Oswestry Disability Questionnaire (ODI) and Numeric Pain Rating Scale (NPRS) in addition to the VISA-G-Norwegian. After one week patients filled out the VISA-G-Norwegian questionnaire once more, without knowledge of their previous score. To ensure that participants had not changed from baseline to retest, they were asked whether, “Their condition had changed since baseline?” Only stable patients answering “no change” were included to the retest arm of the study. All the data were collected on paper, filled out by the patients themselves during the consultations at the hospital.

The VISA-G questionnaire consists of eight questions using a graded response model [1]. The questionnaire addresses pain, everyday activities, physical activities, and difficulty with weight bearing activities. A total score out of 100 points describes the patient’s perceived pain related disability. Lower scores imply severe disability and a higher score indicate less disability [1]. The weighting of question one to seven ranges from zero to ten, while question eight is weighted from zero to thirty. If a participant responded to more than one alternative in questions 1–7, and/or more than one section in question 8, the lowest value was used, as recommended by Fearon et al. [1].

The VISA-G-Norwegian was compared with three outcome measures that are widely used in Norwegian with acceptable psychometric properties: The modified Harris Hip Score (mHHS) [22–24], The Oswestry Disability Index (ODI) [25], and the Numeric Pain Rating Score (NPRS).

The Harris Hip Score is the most widely used PROM for hip prosthesis surgery in Norway [26]. The modified version (i.e. mHHS) is found to correlate largely to the original version of the Harris Hip Score [27]. mHHS excludes the last two of the original items (i.e. the clinical tests) and the total score is multiplied with 1.1 to achieve a scoring from 0 to 100, where 100 is the best outcome, and a score below 70 is considered a poor result. The modified version is used for assessing outcome after total hip replacement, femoral neck fractures and osteoarthritis. It is expected to take 5 min to complete. Studies have shown that it is a reliable, valid and responsive PROM in patients with hip pain [22–24].

The Oswestry Disability Index (ODI) was developed in 1980 to assess pain related disability in patients with low back pain and is widely used today. It includes 10 questions with five verbal response alternatives to yield a total percentage score from 0 (no disability) and 100 (severe disability). If a participant responds to more than one alternative, the highest score is used. ODI has been cross-cultural adapted into Norwegian with acceptable psychometric properties [25].

In a Numeric Pain Rating Score (NPRS) the patients rate their pain intensity from 0 (no pain) and 10 (worst possible pain) [28]. In the present study, participants were asked to rate their average, most and least pain during the last week. The NPRS has shown to correlate highly to other pain scores [29]. A change of two points is considered clinically significant [30].

**Handling of missing values**. There are no written instructions on how to calculate the total score when missing items of the VISA-G. Participants with missing values were excluded from the reliability analysis. For the analyses of validity, participants were excluded if more than 25% of the items were missing for the VISA-G-Norwegian and mHHS. For ODI, percentage is calculated based on the number of answered items.

**Statistical analysis.** Statistical analyses was undertaken using IBM SPSS Statistics for Windows, Version 27.0. Armonk, NY: IBM Corp. Mean and Standard Deviation (SD), median and Interquartile Range (IQR) and frequency (%) were reported according to the scale of the data. Analysis and terminology were chosen based on The COSMIN study design checklist [19]. Correlations were analyzed with Spearman’s rho for nonparametric data.

**Reliability**. Test-retest reliability is the extent to which scores for patients who have not changed are the same for repeated measurements over time [31]. To assess the mean differences in VISA-G-Norwegian score between baseline and re-test, a paired t-test with 95% Confidence Interval (CI) was used. A p-value < 0.05 was considered statistically significant. The relative reliability was assessed with an Intraclass Correlation Coefficient based on a two way-random effects model with absolute agreement (${ICC}_{2.1}$) (${ICC}_{2.1}= \frac{MSBS}{MSBS+ MSE+MSBM}$). An ICC_2.1_ value is given on a range from 0 to 1, where a minimum score of 0.7 was considered acceptable [32, 33]. The variance estimates for the Mean Square Between Subjects (MSBS), the Mean Square Error (MSE) and the Mean Square Between Measurements (MSBM) were obtained from a linear mixed-effects model procedure in SPSS based on restricted maximum likelihood. ICC_2.1_ was calculated for the total score and for each of the items in VISA-G-Norwegian.

While the relative reliability is a measure of the degree to which the measurement differentiates among subjects, the absolute reliability helps us interpret the measure in the same unit as the measurement for individual scores within the subject [34]. The Standard Error of the Measurement (SEM) was used as a parameter of the measurement error and the absolute reliability $({SEM}_{agreement}= \sqrt{MSE+MSBM})$. In addition, the Smallest Detectable Change 95% (SDC_95%_), was calculated as${ SDC}_{95\%}= {SEM}_{ }x 1.96 x \sqrt{2}$. The Bland and Altman plot was used to visually present the measurement error [35].

The internal consistency was examined with a Cronbach’s alpha for each item and the impact of the Cronbach´s alpha if each item was deleted one by one was examined. A Cronbach’s alpha between 0.70 and 0.90 is considered good [33]. Additionally, the item-total correlation was measured for each item and evaluated for exclusion if < 0.3 [32]. Factor analysis on VISA-G has found that a single factor accounts for about 75.1%, thus satisfies COSMINs the assumption of a one dimensional scale for testing internal consistency [18, 19].

**Floor and ceiling effects**. Floor or ceiling effect were considered present if more than 15% of the participants achieved the highest or lowest score for a single item or total score [33].

**Construct validity**. This was assessed with a priori hypotheses, tested through correlations. The hypotheses based on discriminant validity were based on the original development of the VISA-G questionnaire [1]. Thus, VISA-G-Norwegian was correlated to mHHS and ODI. For convergent validity, VISA-G-Norwegian was correlated to NPRS on average since four of the eight questions are asking about disability related to pain.

- Discriminant validity: Small correlation (rho = 0.10 to 0.29) when the VISA-G-Norwegian is correlated to both mHHS and ODI. Indicating that VISA-G-Norwegian is measuring different constructs than both mHHS and ODI.
- Convergent validity: Strong correlation (rho = 0.50 to 1.0) between VISA-G-Norwegian and NPRS on average. Demonstrating that a higher pain on average leads to more activity limitations.

## Results

**Cross-cultural adaption**. Among the 10 patients piloting the VISA-G-Norwegian questionnaire, comments were made about the comprehensibility of question eight. Participants found it difficult to answer due to the design of the question, i.e. they found it confusing to choose between three sections with almost the same wording. To clarify question eight, we did a minor add-on to the layout by including «tick-boxes» to guide and ensure that they only replied to one out of the three response alternatives. In addition to verbal instructions given to the patients before they filled out the questionnaire (to increase comprehensibility). However, to preserve the original constructs of the VISA-G questionnaire it was decided in collaboration with the original developer (A.F.) to keep question eight as in the original version.

**Fig. 1:**
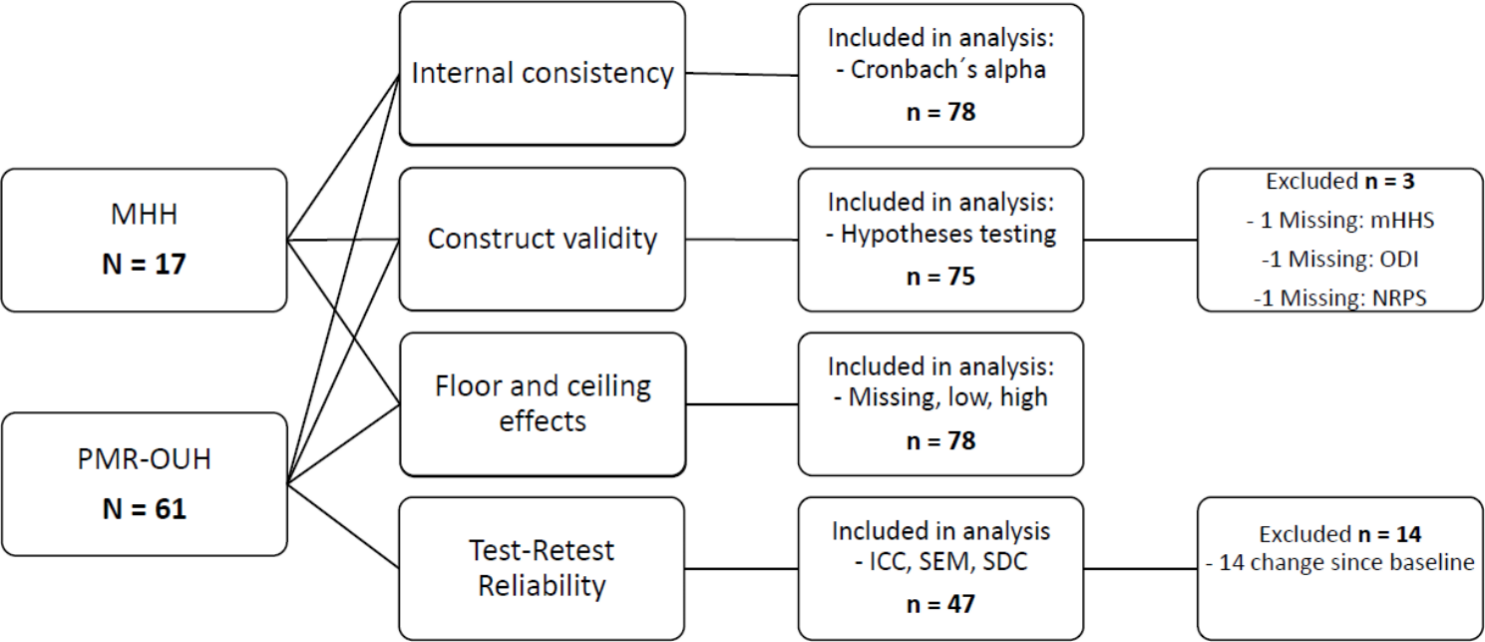
Flowchart for place of recruitment, analysis, inclusion and exclusion. MHH: Martina Hansens Hospital, PMR-OUH: The Department of Physical Medicine and Rehabilitation at Oslo University Hospital, ICC: Intraclass Correlation Coefficient, SEM: The Standard Error of the Measurement, SDC: Smallest Detectable Change, mHHS: modified Harris Hip Score, ODI: Oswestry Disability Questionnaire, NPRS: Numeric Pain Rating Scale

**Participants**. A total of 78 participants were included between November 2019 and August 2022. 83% were female with a mean age of 51 (SD 14) years, 83% had symptoms for more than 12 months, The mean (range) score VISA-G-Norwegian was 55 (16 to 88) (Table 1). 61 participants were included from the outpatient clinic at the Department of Physical Medicine and Rehabilitation at Oslo University Hospital and 17 participants from Martina Hansens Hospital. 47 participants were included for the reliability analyses with 14 participants excluded due to change since baseline (Fig. 1).

**Table 1:** Baseline characteristics of participants

Baseline characteristics	Total cohort n = 78	Test-retest cohort n = 47
Age in years (SD)	51 (14)	47 (15)
Gender female (%)	65 (83)	41 (87)
Main language Norwegian (%)	68 (87)	40 (85)
Symptoms > 12 months (%)	65 (83)	40 (85)
University education (%)	56 (72)	34 (72)
Full time work (%)	39 (50)	24 (51)
Physical activity > 2–3 times a week (%)	60 (77)	37 (79)
Mostly sitting still at work (%)	37 (47)	25 (53)
Problems sleeping > 3 nights a week (%)	30 (38)	19 (40)
Other pain sites (in addition to hip pain)		
Back pain Knee pain	11 (14) 5 (6)	6 (13) 3 (6)
NPRS on average last week (IQR) NPRS on the most last week (IQR) NPRS on the least last week (IQR)	5 (4–7)* 7 (5–8)* 2 (1–4)*	5 (4–7) 7 (5–8) 2 (1–4)
VISA-G-Norwegian total score (0-100) (SD)	55 (17)	57 (17)
mHHS total score (0-100) (IQR)	68 (53–75)*	69 (52–74)
ODI total score (0-100) (IQR)	22 (16–34)*	22 (16–32)

n: number of participants, SD: standard deviation, IQR: Interquartile range, NPRS: Numeric Pain Rating Scale, mHHS: modified Harris Hip Score, ODI: Oswestry Disability Questionnaire, * n = 77

**Reliability**. ICC_2.1_ of the total score was 0.85 (95% CI 0.68, 0.92), the ICC_2.1_ for each individual item ranged from 0.62 to 0.77 (Table 2). There was a 4.3 point difference in the mean VISA-G-Norwegian scores from test to re- test (p < 0.001). With a mean score of 56.5 (SD 17) and 60.8 (SD 16) respectively. The median days from baseline to retest was 7 days (IQR 7–9).

**Table 2:** Results of the relative reliability reported in ICC_2.1_

Item:	ICC_2.1_	95% CI	
1	0.75	0.59	0.85
2	0.77	0.60	0.87
3	0.67	0.47	0.80
4	0.62	0.39	0.77
5	0.75	0.60	0.85
6	0.74	0.57	0.84
7	0.73	0.54	0.84
8	0.63	0.43	0.78
Total score	0.85	0.68	0.92

The reliability ranged by item from a low of 0.62 to a high of 0.77. ICC_2.1_: Intraclass Correlation Coefficient with absolute agreement, CI: Confidence interval

**Fig. 2:**
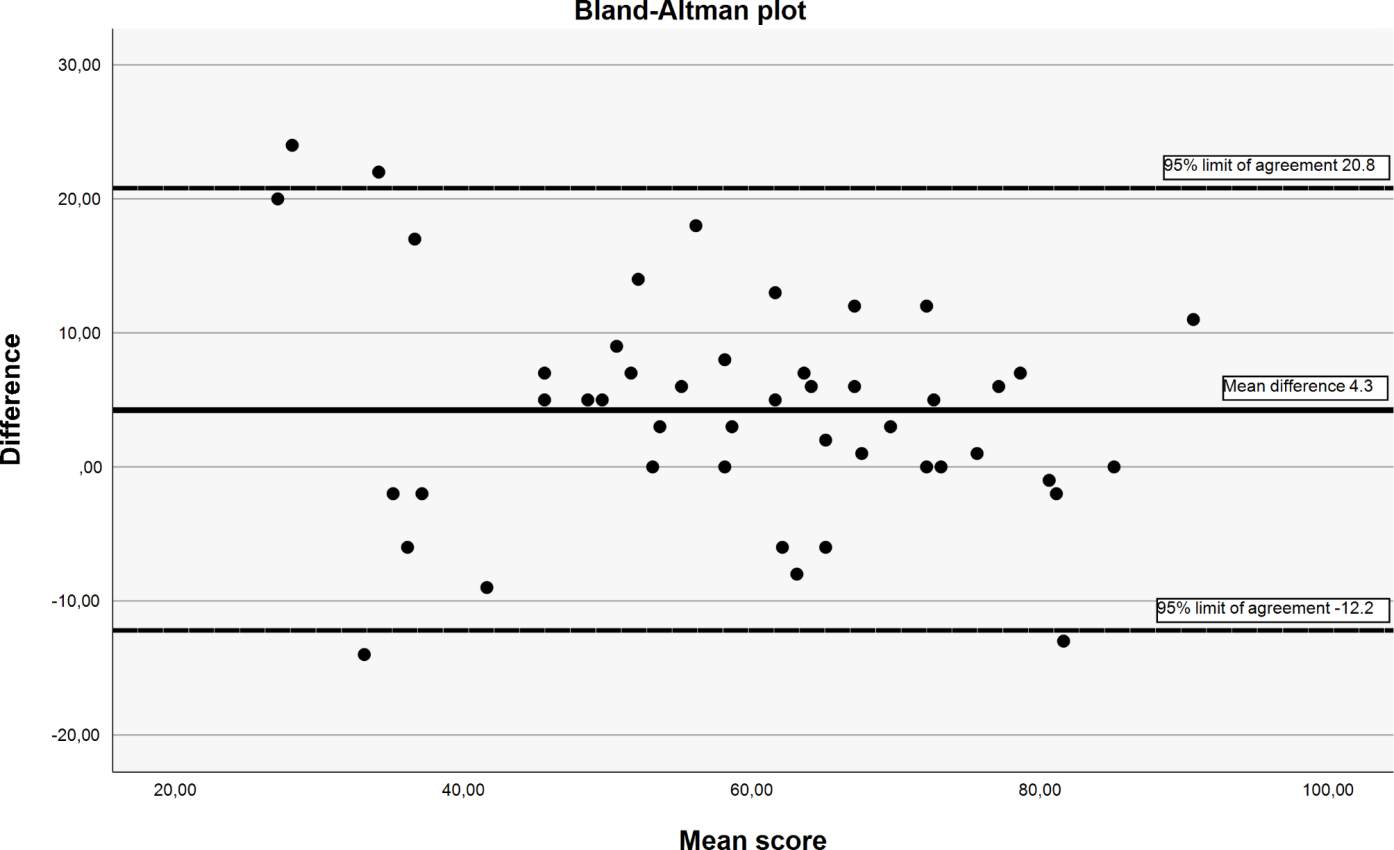
Bland-Altman plot. Illustrating the VISA-G-Norwegian mean difference of 4.3 and the 95% limits of agreement at -12.2, 20.8

${SEM}_{agreement }$ was calculated to 6.6 and the SDC_95%_ was 18.4. The Bland-Altman plot (Fig. 2) showed a mean difference of 4.3 (SD 8.4) and the 95% limits of agreement − 12.2, 20.8.

**Internal consistency**. The Cronbach´s alpha for the VISA-G-Norwegian was 0.77 (95% CI 0.69, 0.84). The highest alpha was detected if item 8 was deleted (0.81), and the lowest if item 6 was deleted (0.71). The item-total correlation ranged from 0.32 to 0.74 (Table 3).

**Table 3:** Internal consistency for the VISA-G-Norwegian

Item:	Item-total correlation	Cronbach´s alpha if item deleted
1	0.62	0.74
2	0.32	0.78
3	0.64	0.74
4	0.68	0.74
5	0.55	0.74
6	0.74	0.71
7	0.50	0.75
8	0.53	0.81

**Floor and ceiling effects**. No floor or ceiling effects were found for the VISA-G-Norwegian total score. However, ceiling effects were found for three of the single items, i.e. item number two (26%), five (23%) and six (44%). Floor effect was only observed for item number two with 22% answered the lowest option. There were no missing items.

**Construct validity**. One of the three hypotheses was confirmed (Table 4). A strong correlation (>-0.50) between VISA-G-Norwegian and NPRS on average was found. The other two hypotheses correlating VISA-G-Norwegian to the mHHS and ODI were not accepted as they had strong correlations.

**Table 4:** Presentation of hypothesis and results

Hypothesis (N = 75)	Spearman´s rho	P value	Hypothesis confirmed
Small correlation (rho = 0.10 to 0.29) between VISA-G-Norwegian and mHHS	0.64	< 0.001	No
Small correlation (rho = 0.10 to 0.29) between VISA-G-Norwegian and ODI	-0.75	< 0.001	No
Strong correlation (rho = 0.50 to 1.0) between VISA-G-Norwegian and NPRS average	-0.63	< 0.001	Yes

mHHS: modified Harris Hip Score, ODI: Oswestry Disability Questionnaire, NPRS: Numeric Pain Rating Scale

## Discussion

The aim of the study was to test the psychometric properties of the VISA-G-Norwegian questionnaire in a population of people with GTPS. The properties were tested according to criteria for good measurement properties, and the study found an acceptable reliability and internal consistency [36]. Hypotheses testing for construct validity found strong correlations to comparator instruments. The sample size of 78 in this study makes this, to our knowledge, the second largest study testing construct validity on symptomatic patients. The only larger study, is the Turkish translation, testing the relative reliability, construct validity and internal consistency with 108 participants [18].

The VISA-G-Norwegian mean score (SD) of 55 (17) at baseline is similar to what was found in the Turkish specialist health care 55 (20) [18]. Comparing the VISA-G-Norwegian mean score at baseline to other GTPS study populations, the mean score of 55 is comparable to other studies reporting about 60 points, while the variation was larger in this study (SD 17 compared to 6 to 11) [14–17, 37, 38]. The slightly lower mean score and the higher variation might be at random or related to the type of participants recruited in a hospital setting in the specialist health care.

For both the relative and absolute reliability, the results were somewhat inferior to what has been previously reported by studies on VISA-G [1, 14–18]. As reported in the Bland-Altman plot, there is less agreement in the lower scores of VISA-G-Norwegian, suggesting a larger measurement error in those with more severe disability. Previous studies have reported ICC_2.1_ between 0.91 and 0.99 [1, 14–18]. These studies have not reported any excluded participants due to change in the condition between baseline and retest [1, 14–18]. In the present study, 14 of the 61 recruited for the reliability analysis were excluded because their symptoms had changed at retest, suggesting that variation in symptoms is likely. In addition, the present study is the only study reporting that the questionnaire where filled out self-administered on paper. E.g., both the Brazilian and French studies reported using phone to collect data, which might have biased results [14, 17].

The SDC_95%_ of 18.4 points suggest that the VISA-G-Norwegian has a large measurement error and therefore a low sensitivity to change. This result is surprising because previous studies have reported a SDC_95%_ from 3 to 12 points [1, 14–17]. However, the observed measurement error in this study is in agreement compared to commonly used questionnaires used by example for patients with persistent subacromial pain in the shoulder [39]. Another contributor to the observed measurement error could be related to the calculation of the questionnaire, especially question eight that can change the score from 0 to 30/100 within the one question.

The pilot study showed poor comprehensibility of question eight. Which presumably might have affected both reliability and validity. However, this study wanted to keep items as close as possible to the original version, for comparability and meta-analysis to already published and future RCTs that uses the VISA-G questionnaire [37, 38]. Deleting question eight in this study increases the Cronbach´s alpha. Item two had a 0.32 item-total correlation, which sits just above the cut-off for exclusion (< 0.3), indicating that the item is measuring a different construct.

This is the first study to investigate floor and ceiling effects on the individual items of the VISA-G. Ceiling effect was found on three of the eight items. This is an important finding as the items is not able to detect change in patients with the lowest or highest score. Ceiling effects at baseline is particularly problematic as there is no room for improvement and responsiveness in the measurement. As ceiling effect is an indication of limited content validity, options of responses for questions with ceiling effects should be considered for revision in a future version of the questionnaire. There were no missing items, indicating that the questionnaire is comprehensible.

The hypotheses for discriminant validity were based on the development of the original VISA-G questionnaire and on the assumption that GTPS related disability could be measured in another construct than pain related disability in low back pain (ODI) or hip joint (mHHS) [1]. After the a priori hypotheses were created, the Turkish, Brazilian and Italian translations published results in contrary to our hypothesis, but in agreement with our correlations, reporting strong correlations of 0.66, -0.77 and − 0.80 between VISA-G and ODI [16–18], suggesting that the questionnaires are measuring the same constructs. The findings of a correlation of 0.20 between VISA-G and ODI in the original development is therefore surprising and not according to the current knowledge about this population. Since non-specific hip and low back pain have considerable overlap in pain related disability, the results suggest that the questionnaires have similar and not different constructs.

### Strengths and limitations

The sample size of symptomatic patients for evaluating the construct validity in this study makes the results more robust than previous studies. Despite being one of the largest studies in this field, only 48 participants were included for the reliability analysis, which is below the COSMIN recommendation of more than 50 for adequate sample size. Participants were included with pragmatic inclusion and exclusion criteria, allowing for a more diverse GTPS population. As many patients with GTPS presents with multifactorial pain, especially in the specialist health care, the results in this study can be considered more generalizable for clinical practice. The questionnaire was only assessed for comprehensibility and relevance in a small pilot. The questionnaire did not undergo a cognitive interview study in the final stage of the translation process as recommended by the COSMIN checklist and was not assessed for comprehensiveness. Thus, may have missed important information about the cultural adaption and possible solutions for the poor comprehensibility of question eight and the low item-total correlation of question two. Since the VISA-G is a diagnosis specific questionnaire, the lack of exclusion criteria resulted in a more heterogeneous study sample which is likely to affect the results, especially the construct validity. Participants underwent an anamnesis and a clinical examination at inclusion, which might have changed their perception of their state, thus, affected their retest.

## Conclusion

The VISA-G-Norwegian has acceptable reliability and validity, despite ceiling effect of individual items. The large SDC_95%_ should be considered when measuring change in similar cohorts with GTPS. For a potential future version, it would be recommended to consider response options for questions with ceiling effect and the comprehensibility of question eight.

## Data Availability

The datasets used and/or analyzed during the current study available from the corresponding author on reasonable request.

## Abbreviations

GTPS: Greater trochanteric pain syndrome
PROM: Patient-Reported Outcome Measurement
ICC_2.1_: The Intraclass Correlation Coefficient
SEM: Standard Error of the Measurement
SDC_95%_: Smallest Detectable Change
VISA-G: The Victorian Institute of Sport Assessment-Gluteal questionnaire
VISA-G-Norwegian: the Norwegian version of the VISA-G questionnaire
COSMIN: COnsensus-based Standards for the selection of health Measurement Instruments
